# A chronological and geographical analysis of personal reports of COVID-19 on Twitter from the UK

**DOI:** 10.1177/20552076221097508

**Published:** 2022-05-05

**Authors:** Su Golder, Ari Z Klein, Arjun Magge, Karen O’Connor, Haitao Cai, Davy Weissenbacher, Graciela Gonzalez-Hernandez

**Affiliations:** 1 Department of Health Sciences, 8748 University of York, York, UK; 2 Department of Biostatistics, Epidemiology, and Informatics, Perelman School of Medicine, 6572 University of Pennsylvania, Philadelphia, PA, USA

**Keywords:** COVID-19, Twitter, social media, prediction models

## Abstract

**Objective:**

Given the uncertainty about the trends and extent of the rapidly evolving COVID-19 outbreak, and the lack of extensive testing in the United Kingdom, our understanding of COVID-19 transmission is limited. We proposed to use Twitter to identify personal reports of COVID-19 to assess whether this data can help inform as a source of data to help us understand and model the transmission and trajectory of COVID-19.

**Methods:**

We used natural language processing and machine learning framework. We collected tweets (excluding retweets) from the Twitter Streaming API that indicate that the user or a member of the user's household had been exposed to COVID-19. The tweets were required to be geo-tagged or have profile location metadata in the UK.

**Results:**

We identified a high level of agreement between personal reports from Twitter and lab-confirmed cases by geographical region in the UK. Temporal analysis indicated that personal reports from Twitter appear up to 2 weeks before UK government lab-confirmed cases are recorded.

**Conclusions:**

Analysis of tweets may indicate trends in COVID-19 in the UK and provide signals of geographical locations where resources may need to be targeted or where regional policies may need to be put in place to further limit the spread of COVID-19. It may also help inform policy makers of the restrictions in lockdown that are most effective or ineffective.

## Introduction

Predicting the spread of COVID-19 is challenging given our lack of understanding in transmission and lack of accurate data to populate prediction models. Tracking and tracing COIVD-19 is imperative in order to inform policy decisions and allocate resources most effectively. Whilst there are numerous online/mobile geographical information systems, dashboards^1^ and applications these systems are often reliant on information from lab-confirmed cases. The lab-confirmed cases that are released daily in the UK were initially only for hospital patients with a medical need before extending to healthcare workers and to more recently to those over 5 s with symptoms who seek a test (https://coronavirus.data.gov.uk/).^2^

There are also delays in the release of these lab-confirmed cases from the first onset of symptoms. Firstly, the patient must seek a test and go to a test centre and then there is the time taken in processing the results (often over 24 h) and then the results need to be collated and released. In addition, not everyone with symptoms will adhere to advice to seek a test or feel well enough to do so.

Initiatives to track the transmission of the virus are becoming available to help overcome these shortcomings. One approach for detecting cases without the need for extensive testing relies on voluntary self-reports of symptoms from the general population.^3^ Initiatives relying on self-reporting of symptoms have included apps such as ‘COVID 19 symptom tracker’ (https://covid.joinzoe.com/) in the UK, surveys disseminated via Facebook (https://jpsm.umd.edu/research/facebook-%28covid%29-symptom-survey), and analysis of posts on social media.^4–7^ These initiatives may all provide useful complementary data to help populate modelling predictions of COVID-19 transmissions.

As of January 2020, there were 16.7 million Twitter users in the UK (https://www.statista.com/statistics/242606/number-of-active-twitter-users-in-selected-countries/). Whilst Twitter users may not be posting to aid symptom monitoring – users often describe symptoms or whether they have or suspect they have COVID-19. Indeed, people often post their symptoms of COVID-19 from the first stages of the disease and do so in real-time (before organizing a test if available or whilst awaiting results). In light of the deficit in testing and the disparities in testing - both temporal and geographical, we propose that social media may provide useful additional insight into symptomatic COVID-19 cases. Using Twitter may also capture people who are reluctant to use an app, fill in a survey or organize a test.

A social media mining approach has already been applied to tweets from users in the United States, China and Italy results.^4–8^ This research has either simply used Twitter volume on COVID-19, or relied on the terms ‘cough’ and ‘fever’ or used synonyms for ‘COVID-19’ as a predictor with surprisingly good results.^4–8^

We propose to automatically analyse the daily trends in the potential exposure to COVID-19 reported on Twitter in different regions in the UK over time. Predicting the contagion and growth by region in the UK is becoming more important as we move towards local confinement rather than blanket lockdowns which are damaging economically, socially and for the nation's non-COVID-19 health issues. To our knowledge, this study is the first use of real-time personal reports in Twitter data from the UK to track COVID-19.

## Methods

The Institutional Review Board (IRB) of the University of Pennsylvania reviewed this study and deemed it to be exempt from human subjects research under Category (4) of Paragraph (b) of the US Code of Federal Regulations Title 45 Section 46.101 for publicly available data sources (45 CFR §46.101(b)(4)).

We aimed to assess the feasibility of using personal reports of symptoms on Twitter to (1) assess whether users report personal information on Twitter that could more broadly indicate potential exposure to COVID-19 or symptoms of the disease, (2) the utility of our social media mining approach for automatically detecting these users and (3) how analysing the chronological and geographical distribution of these reports in the United Kingdom could help timely and effective epidemic monitoring and response.

We used established methods at the Health Language Processing Lab at the University of Pennsylvania (https://healthlanguageprocessing.org/) for systematic collection and semi-automatic analysis of Twitter data. Specific tasks included:

- *Data collection*: Between January and March 2020, we collected more than 7 million publicly available tweets from the Twitter Streaming API that mention keywords related to COVID-19 (such as ‘corona’, ‘coronavirus’, ‘covid’, ‘covid19’ and ‘sarscov2’), are posted in English, are not retweets, and are geo-tagged or have user profile location metadata. We identified approximately 160,000 (2%) of the 7 million tweets that matched handwritten regular expressions (Online Appendix 1) designed to identify tweets indicating that the user potentially has been exposed to COVID-19. We then removed approximately 30,000 (19%) of the 160,000 matching tweets using an automated system for filtering out ‘reported speech’ (e.g. quotations, news headlines) from health-related social media data.^8^
- *Annotation:* Annotation guidelines were developed to help two annotators manually distinguish between three classes of tweets in a random sample of 10,000 of 130,000 of the filtered tweets:
  - Probable: The tweet indicates that the user or a member of the user's household has been diagnosed with, tested for, denied testing for, symptomatic of, or directly exposed to confirmed or presumptive cases of COVID-19. We coded user's as positive who described experiencing symptoms that match those listed as the most common to COVID-19, according to the WHO and the CDC, unless it is ascribed to another reason (e.g. choking on something, smoking, asthma etc.). Symptoms included fever, coughing and shortness of breath or difficulty breathing; and/or the lesser experienced but more unique reported symptoms such as loss of smell (anosmia) or taste (ageusia).
  - Possible: The tweet indicates that the user or a member of the user's household has had experiences that pose a higher risk of exposure to COVID-19 (e.g. recent travelling) or exhibits symptoms that may be, but are less common, associated with COVID-19. Mentions of symptoms that are sometimes present but not the most common symptoms associated with the COVID by WHO and the CDC were annotated as possible cases.
  - Other: The tweet is related to COVID-19 and may discuss topics such as testing, symptoms, travelling or social distancing, but it does not indicate that the user or a member of the user's household may be infected. Mentions of feeling unwell with no specific symptoms mentioned or describing or listing the symptoms of COVID-19 with no indication that the person posting is experiencing those symptoms were classified as ‘Other Mention’ (for more detail: see Online Appendix annotation guidelines).
  The inter-annotator agreement was 0.73 (Cohen's kappa), considered ‘substantial agreement’.^9^
- *Automatic classification.* We split the 10,000 annotated tweets into 80% (8000 tweets) and 20% (2000 tweets) random sets to train and evaluate, respectively, a deep neural network classifier based on bidirectional encoder representations from transformers (BERTs).^10^ After feeding the sequence of tweet tokens to BERT, the encoded representation is passed to a dropout layer (dropping rate of 0.1), followed by a dense layer with two units and a softmax activation, which predicts the class for each tweet. For training, we used Adam optimization with rate decay and warm-up. We used a batch size of 64, training runs for three epochs, and a maximum learning rate of 1 × 10^−4^. Prior to automatic classification, we preprocessed the tweets by normalizing usernames and URLs, and lowercasing the text.
- *City of residence determination*. We used to tweet or profile metadata to derive the user's likely place of residence to the most specific administrative level possible. Although the location of geotagged tweets is commonly assumed to be the place of residence of a user, the percentage of tweets geotagged is very small (estimated at less than 5%). Thus, we utilized a rule-based system to normalize the text provided in the user's profile location metadata.^11^ Additional methods that use the user's publicly available tweets were deployed if necessary. Whilst some posts could only be ascertained as from England, the majority had more specific geographical locations, such as city, county or region. In order to compare the data to that of UK government figures, we coded each city to its appropriate region, for instance, Leeds or West Yorkshire were placed in the ‘Yorkshire and Humber’ region. Those tweets with a US location were analysed in a separate study.^12,13^
- *Data Analysis.* We provide a detailed analysis of those posts with a UK geographical location. We compared the data to lab-confirmed cases over time and per region using cumulative frequency graphs to visually convey the spread of the disease and identify trends in the number of cases.

We did not conduct any statistical comparative analysis on the datasets given that laboratory testing was unavailable during much of the time period studied. Whilst we were unable to identify official social media usage data by region for the UK, we used population data per region as a proxy.

## Results

The BERT-based classifier achieved an *F*_1_-score of 0.64 (precision = 0.69, recall = 0.61) for the ‘probable’ class, 0.53 (precision = 0.54, recall = 0.52) for the ‘possible’ class, and 0.68 (precision = 0.70, recall = 0.67) when the ‘probable’ and ‘possible’ classes were unified, where *F*_1_-score = 2 × recall × precision/recall + precision; precision = true positives/true positives + false positives; and recall = true positives/true positives + false negatives. We deployed the classifier on more than 400,000 tweets, collected between January and April 2020 that match the regular expressions. We identified 4110 cases (58% (2393) probable and (42% (1717) possible) from the UK. The majority were from England (78%, 3206/4110), with 8% (339) from Scotland, 4% (145) from Wales, 1.5% (61) from Northern Ireland and 9% (359) from an unknown country in the UK.

The daily number of probable or possible tweet cases ranged from 3 to 95 with a mean of 45.

**Figure 1.**
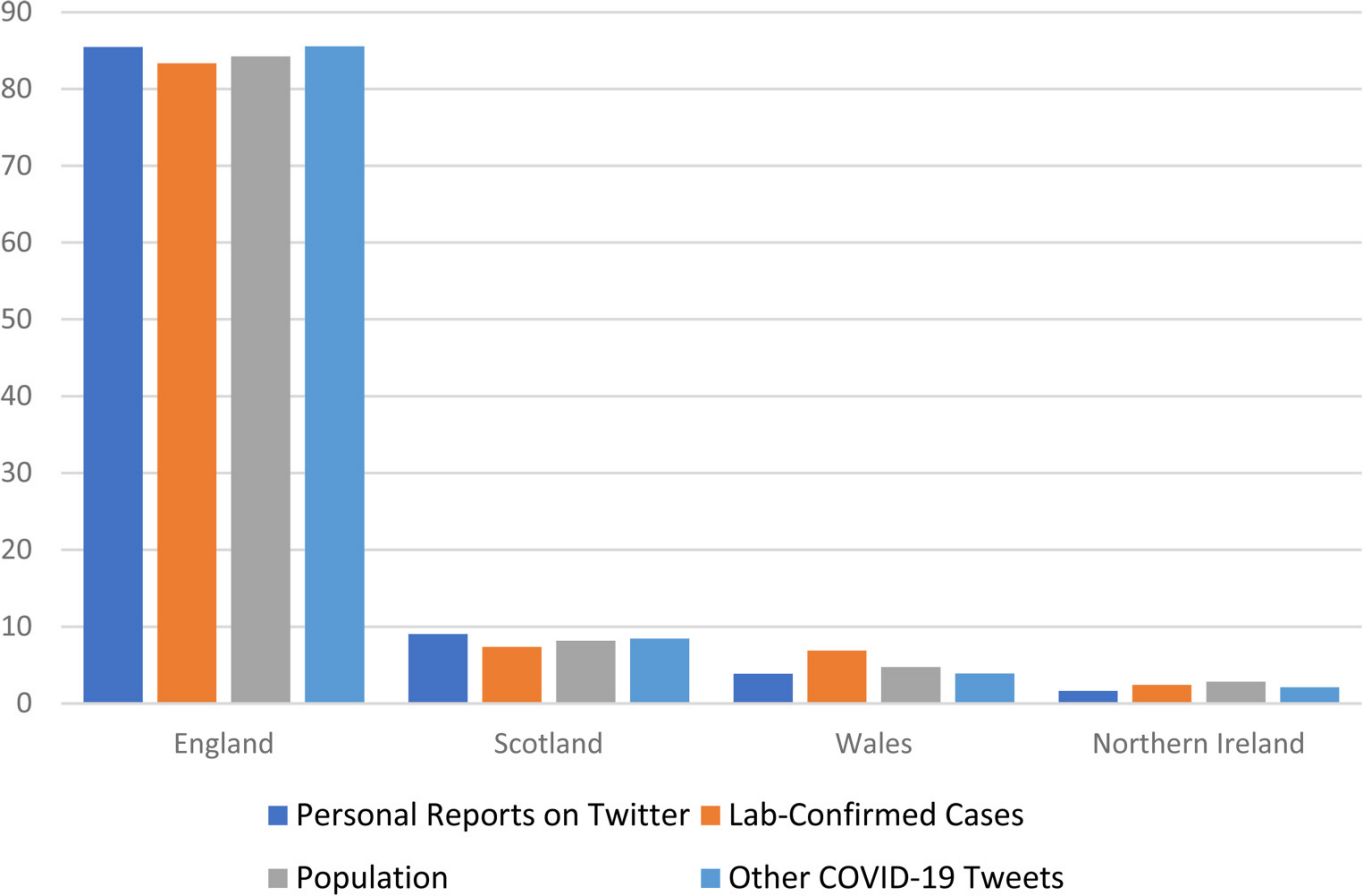
The proportion of users posting ‘probable’ or ‘possible’ tweets by UK country compared to the proportion of lab-confirmed cases, the proportion of the population, and the proportion of other COVID-19 tweets per country, 23 January 2020 to 28 April 2020. These data are based on 3751 ‘Personal Reports on Twitter’, and 145,634 ‘Lab-Confirmed Cases’ from https://coronavirus.data.gov.uk/, a population of 66,435,550 from https://www.ons.gov.uk/peoplepopulationandcommunity/populationandmigration/populationestimates/datasets/populationestimatesforukenglandandwalesscotlandandnorthernireland and a total of 41,350 other tweets.

Figure 1 illustrates the number of detected users from each nation in the UK who have posted ‘probable’ or ‘possible’ tweets between 23 January 2020 and 28 April 2020 and compares this to; the UK government statistics for lab-confirmed cases by nation, the population of each nation and the proportion of other COVID-19 tweets retrieved from each nation. This figure clearly indicates that for the different nations in the UK the figures are consistent between the personal reports on Twitter and lab-confirmed cases. However, Wales and Northern Ireland appear to either tweet slightly less or we have not retrieved quite as many of their tweets (this could be due to language differences).

**Figure 2.**
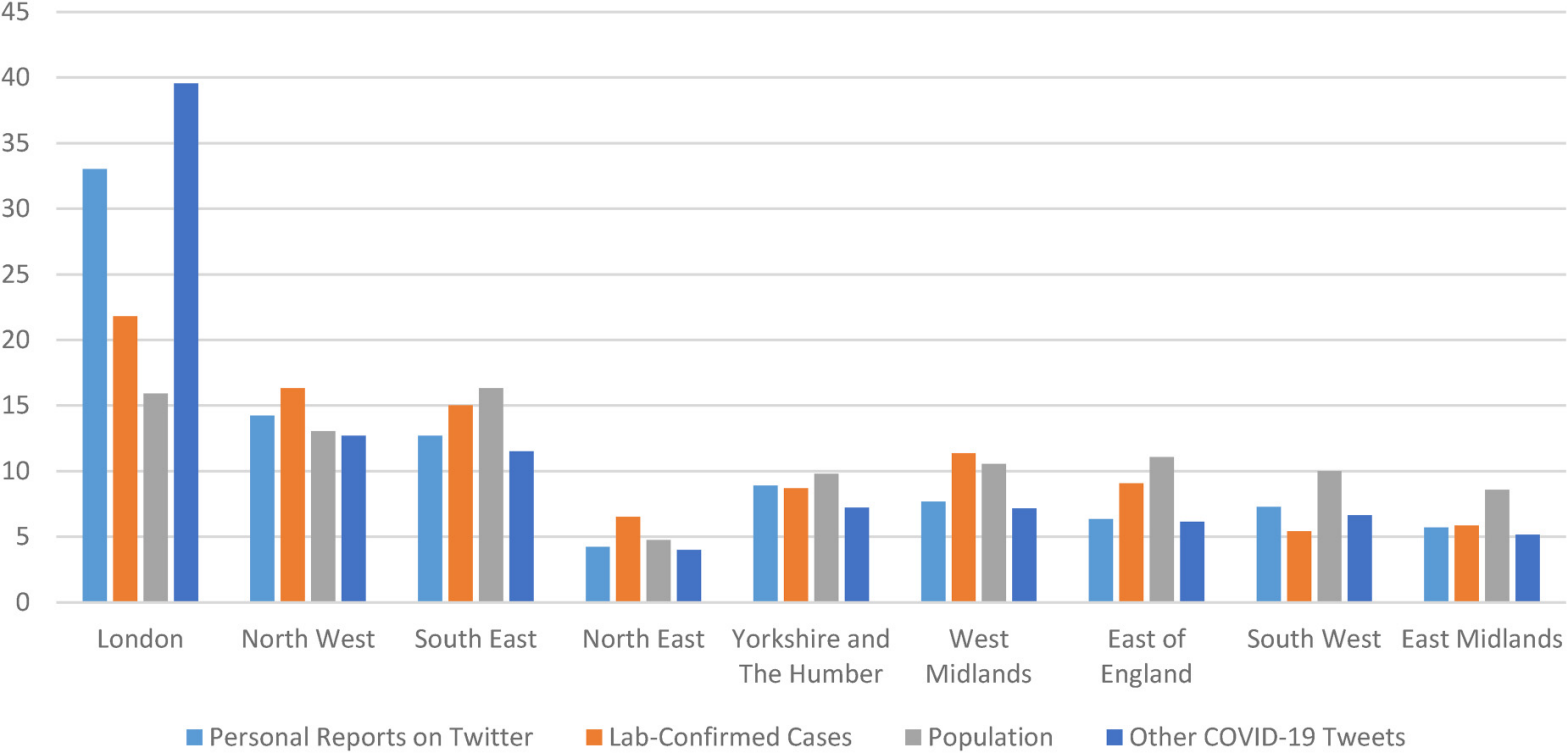
The proportion of users posting ‘probable’ or ‘possible’ tweets by region in England compared to the proportion of lab-confirmed cases, the proportion of the population, and the proportion of other COVID-19 tweets per region, 23 January 2020 to 28 April 2020. These data are based upon 2917 ‘Personal Reports on Twitter with a regional location within England’, 11,0385 ‘Lab-Confirmed Cases in England’ from https://coronavirus.data.gov.uk/, a population of 55,977,178 from https://www.ons.gov.uk/peoplepopulationandcommunity/populationandmigration/populationestimates/datasets/populationestimatesforukenglandandwalesscotlandandnorthernireland and a total of 32276 ‘other tweets’.

Figure 2 illustrates the proportion of users posting ‘probable’ or ‘possible’ tweets by region in England compared to the proportion of lab-confirmed cases, the proportion of population per region and proportion of other COVID-19 tweets per region, 23 January 2020 to 28 April 2020. There is a notably high number of tweets from London, not just personal reports but also of other COVID-19 tweets. This may reflect a younger demographic in London who are tweeting more than outside London. The other regions appear to have an overall agreement with the lab-confirmed cases. It should be noted that the lab-confirmed cases are not necessarily an accurate reflection of actual cases given the limited testing available in England. This limitation applies to all regions, however.

**Figure 3.**
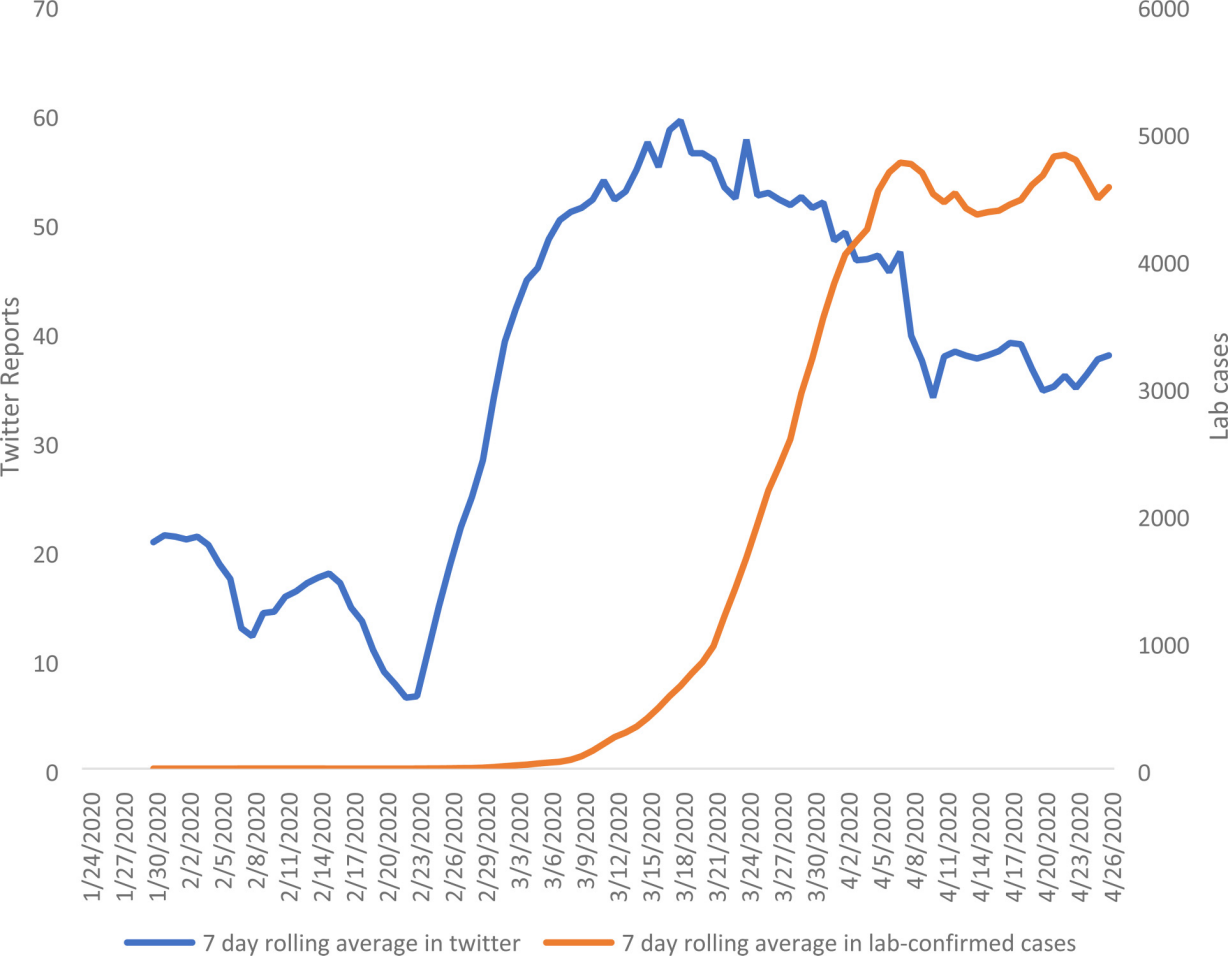
Seven-day rolling average of number of users posting ‘probable’ or ‘possible’ tweets, and cumulative lab-confirmed cases, 23 January 2020 to 28 April 2020 in the UK. Footnotes: Data for testing carried out is only released from 31 March 2020. The seven-day rolling average increased steadily from 13,598 on 4 April 2020 to 28,304 on 26 April 2020 https://coronavirus.data.gov.uk/. There is a gap in data collection of tweets from 4 to 7 April 2020.

Figure 3 illustrates the seven-day rolling average of users posting ‘probable’ or ‘possible’ tweets, and cumulative lab-confirmed cases, from 23 January 2020 to 28 April 2020. When comparing the curve of the personal reports on Twitter and lab-confirmed cases we can see that the spike appears earlier in the tweets than in the lab-confirmed cases. The shape of the cumulative curve indicates whether the daily number of cases is increasing, decreasing or staying the same. Both graphs demonstrate a similar pattern of spread of COVID-19, however, lab testing was unavailable in January and February and extremely limited in March 2020.

**Figure 4.**
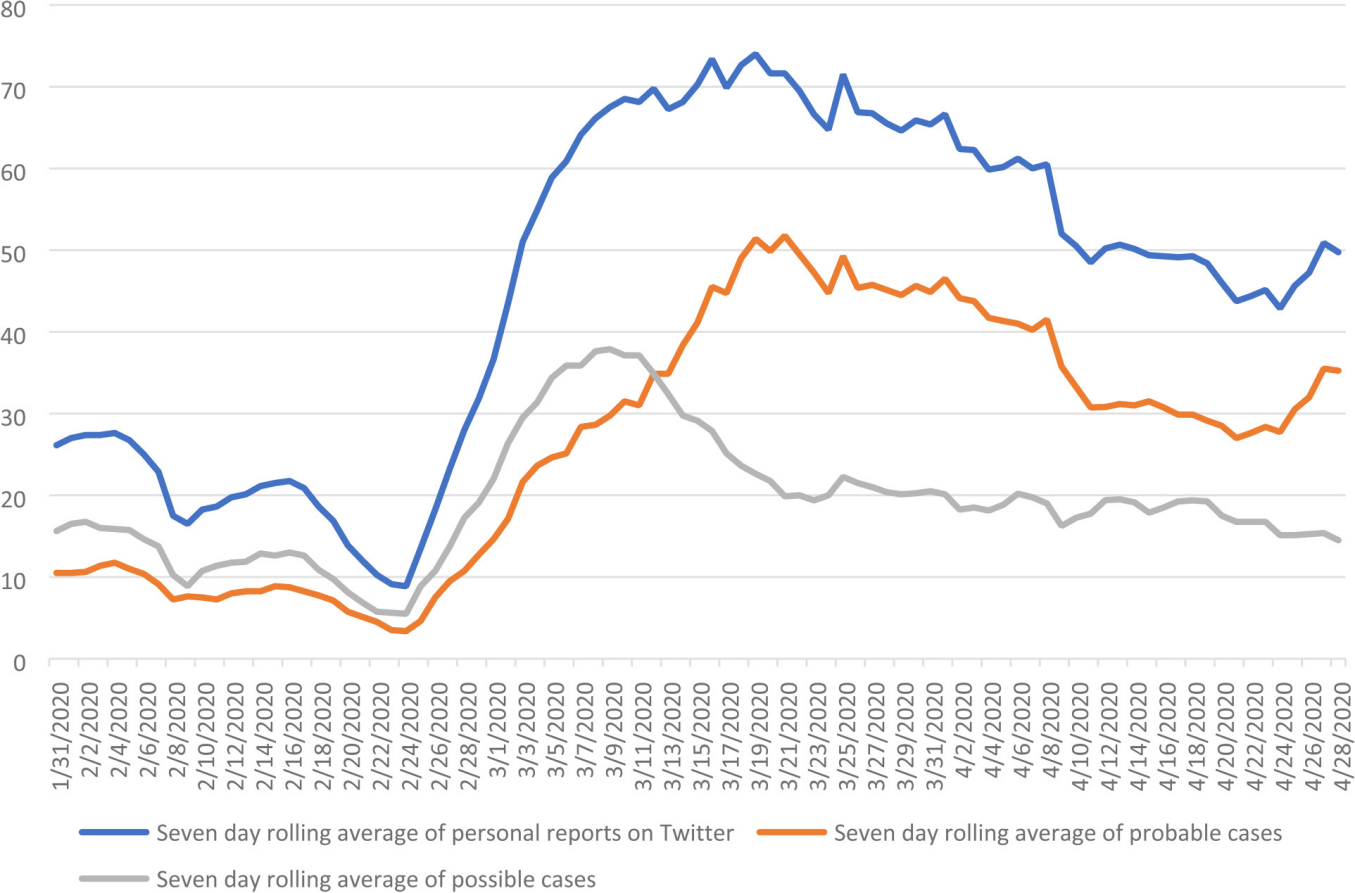
The seven-day rolling average number of users posting ‘probable’ or ‘possible’ tweets, and personal reports on Twitter, 23 January 2020 to 28 April 2020 in the UK.

Figure 4 illustrates the seven-day rolling average number of users posting ‘probable’ tweets, ‘possible’ tweets, and the total cumulative number of personal reports on Twitter, from 23 January 2020 to 28 April 2020. This demonstrates the increasing proportion of tweets that are probable as opposed to possible cases.

## Discussion

In Figures 1 and 2, there is an overall agreement in the proportion of personal reports on Twitter at a national and regional level to those from UK government statistics of lab-confirmed cases. In the future, the analysis could be more detailed by assessing trends in smaller geographical areas as much of the data contained information at the city or town level or borough in the case of London.

Figure 3 indicates that personal reports on Twitter began to increase sharply around the beginning of March but not until the middle/end of March for UK government confirmed cases or deaths. The comparison of the figures shows some agreement in trends but more reports were on Twitter when no testing was available. The lab testing may also show a higher rate of increase as more testing became available.

Figure 4 suggests that tweets had more clarity in their content as time progressed and were more likely to report the common symptoms of COVID-19 as the proportion of probable as opposed to possible cases increased.

We have detected ‘probable’ or ‘possible’ tweets, therefore, that were posted before the first confirmed case in many regions. This raises more questions than answers but is an interesting finding given that the first confirmed cases and first confirmed deaths are now thought to be early than first recorded. Thus, our research suggests that real-time, user-generated Twitter data could help provide early warning signals of the spread of COVID-19. This finding is similar to other research on social which indicates that Twitter may have the capacity to detect formal outbreaks up to 7 days ahead^14^ or even 7 to 19 days ahead of official recordings.^4^

Other studies have focused on the US to ascertain case estimations of COVID-19, with similar results to our study in the UK.^13,15,16^ To the best of our knowledge, our study is the first to examine the use of social media data in the UK. Other UK studies have focused on online search behavior^17^ or online apps.^18,19^

Monitoring COVID-19 outbreaks are not the only potential use of social media data in the COVID-19 pandemic. For example, by using social media data, researchers were able to identify an emerging a spectrum of symptoms, such as anosmia and ageusia, body aches and skin lesions,^20–22^ and to identify common combinations of symptoms earlier than in the biomedical literature.^23^ Other studies have used social media to conduct more qualitative research on public views and opinions.^24–26^

A limitation of our study is in the comparison of the twitter cases to lab-confirmed testing. We are unable to conclude whether the trends observed are causally related.

We need more detailed research into how our approach to scrutinizing Twitter posts can help predict the spread of COVID-19 and how such predictions may help monitor the situation going forward, particularly in light of the lifting of some restrictions on lockdown and the plan for more regional lockdowns.

## Conclusion

Twitter posts may indicate COVID-19 peaks before the results of government lab-confirmed cases are released and with geolocations available for many tweets this can indicate geographical trends throughout the UK. This will be particularly useful to inform policy at a national and local level.

## Supplementary Material

Supplementary material

Supplementary material
