# Supplementary material for: A chronological and geographical analysis of personal reports of COVID-19 on Twitter from the UK
Source: Digit Health. 2022 May 5;8:20552076221097508. doi: 10.1177/20552076221097508 (PMC9096830; doi:10.1177/20552076221097508)
Supplement: Supplementary material [file sj-pdf-2-dhj-10.1177_20552076221097508.pdf]

ANNOTATION GUIDELINE FOR CORONAVIRUS TWEET CLASSIFICATION

Health Language Processing Lab
Department of Biostatistics, Epidemiology and Informatics
University of Pennsylvania

Guideline Revision Information

Author	Version	Date	Changes
Karen O'Connor	0.1	3/20/2020	Initial Draft
Karen O'Connor; Ari Klein	0.2	3/23/2020	Added a third classification type for possible exposure
Karen O'Connor	0.3	3/25/2020	Clarified 'possible' category; added examples

Introduction

In this annotation project, we are interested in classifying tweets as indicating that the user, or member of their household, has been exposed to the Coronavirus or has contracted or is experiencing common symptoms of COVID-19 (“Probable Case”), instances where the user indicates that they were in a situation where it may be possible they have been exposed, or had possible contact with a confirmed or suspected case, or are exhibiting some possible symptoms (“Possible Case”) or not having any such indications (“Other Mention”). Our corpus consists of tweets that contain the mention of a certain keywords related to coronavirus. These include, <fill in keywords>. Each tweet will be classified as either a Probable Case, Possible Case, or Other Mention, based on the information in the tweet. The purpose of this document is to define the indicators of each class and describe the criteria that the annotators should use to determine whether the tweet is a Probable Case, Possible Case, or Other Mention. The annotated data will be used to train automated classification systems.

The annotation guidelines are an evolving document and changes and updates will be made over time. All updates will be noted and dated in the Guideline Revision Information section

Annotation Tool

For these annotations, we will use an Excel spreadsheet. The spreadsheet will contain certain information about each tweet such as userId, tweetID, date, drug name and the tweet text. Annotator will have a column to place the appropriate code (0=Other Mention, 1=Probable Case, 2= Possible Case). Additionally, there is a “Notes” column for the annotator, defined in the following section.

General Guidelines

Each tweet should be classified with only one code.

The ‘Notes’ column is not required to be used but is there for annotators to place any comments or notes that they have about annotating that tweet.

For this study we will consider not only the user (person tweeting) but also discussions about members of their households when determining the correct class annotations. Household members include spouses, children at home, roommates and any other relative (eg, parent, aunt, cousin) if it can be determined that they reside in the same household.

The rest of the guidelines will define and describe for annotators each class and indicators in the tweets that can be used to assist in determining the correct classification.

Probable Cases

Probable cases are those that indicate that the user, or a member of their household:

- has contracted Coronavirus disease (COVID-19) and/or expresses that he/she has been tested or diagnosed with it or;
- self-diagnoses as having Coronavirus disease (COVID-19) **and** is symptomatic or;
- expresses having been directly exposed to Coronavirus but is asymptomatic

There are several indicators, or topics of discussion, that should be considered by the annotator when determining if the tweet should be classified as a Probable Case including diagnosis, testing for the virus,

experience of symptoms, direct exposure to someone with confirmed or suspected COVID-19. While some tweets may contain more than one indicator, only one is needed to classify the tweet as positive.

Diagnosis

The user states that they, or a member of their household, have been diagnosed with, or are recovering from, COVID-19.

i. *I just **tested positive to the corona virus**. I'm too weak to even feel sorry for myself, but I intend to share my symptoms to allow people quickly identify and self-isolate immediately, to avoid infecting others.*

ii. *I'm **recovering from Covid-19 infection**, why aren't they're more stories in the media about the recovery and demographics of who is getting sick?*

Testing

The user discussing getting tested or wanting to get tested for themselves or a household member. For these tweets, we will assume that the user, or their household member is seeking testing due to being exposed or symptomatic, even in the absence of such a situation being mentioned in the tweet. Tweets discussing testing should be classified as Probable regardless of whether the person was able to obtain testing or has received the results of the testing. However, if the user states they were tested and the test was negative, then the tweet should be classified as Other Mention (see: Other Mention: Testing)

iii. *Cue the "Benny Hill" theme... 👁👇 @acarvin: Last Thursday I was admitted to the ER w/ #coronavirus symptoms, including chronic cough, shortness of breath &amg pain. I was **given a #COVID19 test** and told I'd have the results within 48 hrs. https://t.co/bwgMswn72m*

iv. *Do I have **corona**? Idk they **won't test** me. But whatever I have Probably isn'**t** gunna kill me and that's disappointing.*

In (iii), the user is stating that they have been tested and are awaiting results. In (iv), the user has been refused testing and though we cannot infer whether they have a legitimate reason for wanting testing, we will mark these cases as positive potential cases.

Symptoms

User describing experiencing symptoms that match those listed as the most common to COVID-19, according to the WHO and the CDC, including fever, coughing and shortness of breath or difficulty breathing; and/or lesser experienced but more unique, reported symptoms such as loss of smell (anosmia) or taste (ageusia). Additionally, users who state that they have pneumonia or flu-like symptoms and/or have tested negative for these should also be coded as positive.

Mentions of symptoms that are sometimes present but not the most common symptoms associated with the disease listed above, should be annotated as possible (see: Possible: symptoms)

v. *day 3 of having a **fever**, **dry cough** & **sore throat**. i tested **negative for flu** & strep but was refused to test for covid-19 because i'm a "low risk"*

vi. *this country is PATHETIC. i'm having a fever, cough, migraine, etc and they won't give me a **test** for **covid**. i tested **negative** for the **flu**. still no **test**. THEY DIDNT EVEN LISTEN TO MY LUNGS LMAOOOOOOO*

vii. *My wife has been sick with a persistent cough, so we have self-isolated. I've been fine, except I've **completely lost my sense of smell**. Upside - can't even smell poopy diapers. This, apparently, is a suspected symptom of COVID. I didn't know, so I'm telling you. Stay home.*

In (v) & (vi), the user is stating signs of infection as well as a negative result for a flu test. In (vii), the user mentions one of the unique symptoms of the disease.

Single main symptom mention

While cough, fever or shortness of breath mentioned on their own can be attributed to other diseases, as they listed as one of the main symptoms by the CDC and WHO, we will define their mention, even in the absence of mentions of other symptoms, as an indicator of the positive class unless it is ascribed to another reason (eg, choking on something, smoking, asthma, etc.) (See: Other Mention: Symptoms).

xi. *Everybody's scared over nothing, I know I'm not getting Coronavirus. I just got a light **cough**.*

xii. *A morning of incessant **coughing** and sending four emails and I'm exhausted. These crap lungs will be the death of me even before Coronavirus hunts me down....*

Mentions having flu or pneumonia

Given the similarity of symptoms, if the user mentions that they, or a member of their household, has the flu or pneumonia but there is no indication that they have been tested for either and/or it is possible they are self-diagnosing, these tweets should be classified as positive.

xiii. *I just went home from work early because I thought I was running a fever. I'm not - I basically **just have a mild case of the flu**. Fucking coronavirus is just screwing with me...*
xiv. *the coronavirus has me insecure about coughing, bruv i promise **it's just the flu** 🤧*
xv. *@BradleyJames today spike channel gifted us with some #Merlin.ep. just wanted to say thanks for the laughters. My family & I live in Veneto we re **sick at home because kids caught flue** at school which is NOT coronavirus still situation is what it is .For 1 h we lived in the magic!*
xvi. *When you **have pneumonia** and they STILL won't test you for corona*

Mentions of past illness with similar symptoms

Users who mention having had symptoms that match those of COVID-19 in the recent past (ie, November to January) but before it there was widespread testing should be annotated as positive

viii. @RichardEngel I have a serious, yet possibly ignorant, question. How do we know that the Coronavirus is just now appearing in the US? My hubs & I were both **very ill in Jan**. & doctors didn't know with what! We **had flu-like symptoms yet tested negative for flu/strep**.
ix. I don't mean to seem glib or self-centred but I had a bad virus **in November that came back in December and had all the Coronavirus symptoms**. Never had such a bad dry cough and I had the shortness of breath and hot sweats as well?

Direct Contact with Diagnosed/Suspected COVID-19 patient

The user states they, or a household member, have been in prolonged contact with a patient who has/or is suspected to have COVID-19. The annotator can infer contact in instances of a close family member being mentioned, eg, spouse, child, or of someone where the probability they have interacted recently may be high, such as a co-worker.

x. *Absolute joke!Me, Marc & Florence self isolating for 2 weeks even though we're all fine but* ***been in contact with someone with suspected corona*** *on Sunday.****Won't test*** *me even though im a nurse and causing my work strain at already a difficult time! Why hell* ***won't*** *they just* ***test*** *me?!*

xi. *My* ***coworker most likely has #coronavirus****. He and his wife are presenting with all the symptoms: dry cough, high fever. He* ***sits in the office next to me****. Here's the kicker -- he and his wife went to a hospital to get tested, but they refused to test him. We're not ready for this.*

xii. *Today* ***2 ppl got quarantined on our base for Coronavirus*** *and of course on* ***Tuesday my husband was sharing food with one of them****...*

xiii. My **roommates is currently coughing** a lot and throwing up, and blowing his nose a lot. This #coronavirus may be more real then I thought time to suit up. https://t.co/MSC7ZAt2CV

Self-Isolating/Self-Quarantine

The person states that they are in insolation or quarantine due to the possibility of having contracted or knowingly being exposed to the virus

xiv. *Gen Z here, almost everyone in my family has been in contact with someone who was diagnosed with COVID-19, so I'm under strict* ***quarantine*** *and I couldn't be happier.*

Possible Cases

Possible Cases are those that indicate that the user, or a member of their household:

- has been in a situation or place with a higher probability of exposure to Coronavirus, or
- mentions that someone near them in a confined space was exhibiting possible symptoms of COVID-19, or
- is experiencing symptoms that may be present with the disease, however these symptoms are not listed as the most common symptoms by WHO and the CDC

These are cases where the user, or a member of their household, were in a situation with increased risk for exposure or are exhibiting signs of some illness, however, there little confirmatory evidence present in the tweet that they were definitely exposed to the virus. As such, the evidence in the tweet may not be as strong as those categorized as Probable cases. There are several indicators or topics of discussion that should be classified as Possible Cases including traveling by public transportations, or visiting a doctor's office or hospital and/or being in the presence of someone exhibiting signs of sickness, exposure to someone who should be in quarantine even with no mention of that person exhibiting symptoms, or the user talks about someone with confirmed or suspected COVID-19, however, it is not clear that the user has been in recent close contact with that person.

Travel

The user states that they or a member in their household are, or have recently been traveling, such as by airplane, cruise ship or train, including public transportation. For these, there should be evidence that the person actually traveled and is not discussing future plans (see: Other Mention: Travel).

i. ***Disembarked a flight*** *this a.m Denpasar>Mel (Tul) & spent at least 35 mins inescapably rubbing shoulders with many families arriving from all over #China. Mark my words, the airport is waiting to claim it's first #coronavirus victim if it hasn't already. #publichealth*

ii. *We haven't got a bloody chance of containing this virus. Half of* ***my packed train*** *carriage is sneezing and sniffing and I haven't seen a single tissue or handkerchief. #CoronaVirus*

iii. *Customs officer apologized on the way out for the wait. Said that passengers had been under evaluation since* ***we got off the plane*** *but that there was no communication between the various agencies within the airport monitoring/screening passengers @THV11 #coronavirus #TravelBan*

iv. *This whole coronavirus is a tad bit concerning because all I do is fly and work in hospitals in larger cities. I've* ***been in Miami, DC and Atlanta (2x) this week****. So I'm probably gonna get it*

v. *My* ***wife's flight*** *from Atlanta to Boston this morning before and after boarding.* 😳 *#peoplefreakinout #coronavirus #COVID19 https://t.co/xCOJWuIlMB*

Self-Isolating/Self-Quarantine due to travel

The person states that they are in insolation or quarantine due to travel, however they do not state having any symptoms or having come into contact with a positive case

vi. *Managed to get on what seems to have been* ***one of the last flights out of Italy*** *last night. Utterly surprised our flight was NOT seperated from the other approx 10 flight arrivals at passport control...but yet I now have to* ***self isolate*** *for 2 weeks! @BristolAirport? #coronavirus*

Symptoms

User describing symptoms that they are worried are COVID-19 however the symptoms are not one of those most commonly associated with the disease or could have a variety of other causes, such as fatigue, nausea, body aches, sore throat, headache, and gastrointestinal issues, or the symptoms have been present for a period of time without the development of other symptoms.

vii. *Tbh, This worldwide Coronavirus pandemic is scary as shit. One of my* ***throat glands R sore****, struck me 2 days ago. But other than that & my usual allergies, Im perfectly fine & feel totally well, and I haven't traveled outside the country for well over a year. Should I get tested?*

viii. *I'm still sick ... and this coronavirus stuff is making me nervous ... □□* ***my upper respiratory infection is now a lower respiratory infection*** *per usual how my body handles getting sick. Always in my lungs. Always ....*

Mentions of feeling unwell with no specific symptoms mentioned should be classified as Other Mention (see: Other Mention: Symptoms)

Testing Positive for Flu or Pneumonia

Given the similarity in symptoms and the fact that it is unclear whether a person can simultaneously be infected with both disease, tweets mentioning that the user, or a member of their household has tested positive for the flue of pneumonia should be classifies as possible.

xi. *@Jaz_Barton @davidsirota She went to the ER, they refuse to test her. Stated she doesn't meet the criteria. I argued with them, telling them you can't tell me she doesn't have Coronavirus because she tested positive for the flu. In what medical journal does it say that! This virus is too new!!*

Possible Exposure to Diagnosed/Suspected COVID-19 patient or someone exhibiting symptoms

Unknown Contact

The user states they know someone who has/or is suspected to have COVID-19, however, it cannot be inferred from the tweet whether the user has had contact with that person or not

ix. *My **friend's experience trying to get tested for coronavirus** (COVID-19) today. Warning: Will not alleviate fears of spread 😷 https://t.co/NseboB4Ltx*

x. *someone who **lives at my apartment complex got diagnosed with the coronavirus**... is this a good time to ask for some free rent?*

xi. *UK strategy on Coronavirus seems bad. I **know someone with symptoms** who works in a high-contact job in London who was told by NHS she won't be tested and should continue going to work because she hadn't returned from hotspot. That's bad advice even if she just has the normal flu!*

Unknown Disease State

The user is in a place where close quarters increase the chance of contracting the virus, however, the person they have been in contact with is exhibiting some symptoms but the cause of the symptoms unknown or conjecture by the user.

xii. *@uhdowntown There was a kid in the business building 2nd floor computer lab around 1:00pm wearing a yellow shirt. Someone needs to ask him if he is okay. He is super sick and I **think he has coronavirus** or definitely the flu. He is Asian. And I mean he is sick sick. Like super sick. I left.*

xiii. *An elderly woman just **sat down 2 chairs from me** in the airport terminal and proceeded to **seriously cough** up her lungs. I got up and moved, but I'm concerned she is on my flight. Why arent we screening for super sick passengers? #coronavirus*

Indirect Contact

The user had indirect contact with a suspected case of COVID-19.

xiv. *Lmao this was my flight ... we had our entire cleaning team quit and refuse to clean the plane after we heard the PAX possibly could have coronavirus. We (customer service agents) ended up cleaning and we could possibly end up having to be quarantine 😌 will update*

Direct Contact with Someone who may have been exposed

The user is in contact with a person who has a higher risk of exposure due to their recent activity but there is no confirmation that the other person was exposed

xv. *My teacher just came back from Italy and should be in quarantine was back in front of the classroom and now they are out sick*

Other Mention

Other Mention cases include discussions about the Coronavirus and COVID-19 but do not relate to the health or the user or anyone in their household,

General Discussions about Coronavirus or COVID-19

Tweets that just mention the virus or disease, such as news reports, opinions or jokes, should be classified as negative.

Examples:

i. *US chartering flight to evacuate American diplomats and citizens out of China amid coronavirus outbreak https://t.co/yKUwZOFmJ3*
ii. *just came back from dinner in Flushing so I'll let you know if I have coronavirus* 🙃
iii. *Every time I read the news, I feel short of breath. No cough or fever yet though. Am I infected??? #COVID19 #coronavirus*
iv. *I think me and my friends have CoronaVirus and we are in a Pub. Can we remain here and be quarantined for 14days? Cyril? https://t.co/3pbtqJQOuH*

These general discussions may also touch on subjects that were indicators under the Probable or Possible class, however, if these are just general and not related to the person contracting or possibly contracting COVID-19 then they should be classified as Other Mention.

Some examples of Other Mention cases:

Testing

General discussions about testing or lack thereof with no indications that the user need to or is trying to be tested:

v. *So I jus wanna let everyone know that even in all this panic that in gulf shores Alabama they **won't** even **test** you for **corona** if you haven'**t** been outta the country, how are we supposed to be as safe as we can if we can'**t** even be diagnosed free testing should be given TO EVERYONE*
vi. *It sucks that they **won't** give people the **corona** virus **test** unless you fit the criteria. So people who are not showing any symptoms or symptoms that aren't deemed extreme, you **won't** get the **test**.*

Tweets that discuss the user receiving a test and that test returned a negative result should be annotated as Other Mention

vii. *Seven days later ... I got my result. I tested negative for covid!*

Symptoms

Tweets that are describing or listing the symptoms of COVID-19 but give no indication that the person posting is experiencing those symptoms:

viii. *we be playing bout the coronavirus like if we ain't gone lose our shit the minute we start sneezing & coughing.* 😭😭😭😭

Tweets where the user states non-specific symptoms, such as feeling sick, or a symptom not associated with COVID-19, such as sneezing:

ix. *if you see me with a face mask at church , no... i don't have the damn coronavirus . i'm just sick and i'm not trying to get other people sick* 🙄

x. *Ended up going home really sick* 😖*today. I'm worried I might have some sort of virus. I seriously doubt it's the Coronavirus*

Tweets discussing other people exhibiting possible symptoms but it is not evident that the user is in close contact with those people:

xi. *Y'all need to stop coughing without covering your mouths because I don't want the coronavirus*

Tweets that discuss the user is exhibiting one of the main symptoms but attributes its cause to a non-health related, or other underlying health condition:

xii. *I started coughing because I choked on my water but everyone lookin at me like I got coronavirus*

Travel

Any travel that is being planned or has not yet occurred:

xiii. *I hope this coronavirus scare doesn't ruin my cruise next month*

Self-Isolating/Self-Quarantine

User is discussing being in quarantine or isolation due to general recommendations of social distancing or shelter in place orders, but not due to having been in contact with anyone positive for COVID and showing no symptoms.

xiv. *I social distanced very good today. Didn't leave home once. #SocialDistancing #StayTheFHome #coronavirus #COVID19*

xv. *I could think of worse things than being quarantined for 14 days for our own safety. Like....getting the coronavirus. We'll all still have our devices. We'll all still have Netflix. Not that big a deal.*
